# A mixed methods study to adapt and implement integrated mental healthcare for children with autism spectrum disorder

**DOI:** 10.1186/s40814-019-0434-5

**Published:** 2019-03-28

**Authors:** Nicole A. Stadnick, Lauren Brookman-Frazee, David S. Mandell, Cynthia L. Kuelbs, Karen J. Coleman, Timothy Sahms, Gregory A. Aarons

**Affiliations:** 1 Department of Psychiatry, University of California, San Diego, La Jolla USA; 2 Child and Adolescent Services Research Center, San Diego, USA; 3 Department of Psychiatry, Center for Mental Health, University of Pennsylvania, Philadelphia, USA; 4 Department of Research and Evaluation, Kaiser Permanente Southern California, Pasadena, USA; 5 Department of Pediatrics, University of California, San Diego, La Jolla USA; 6 Rady Children’s Hospital, San Diego, USA; 7 San Ysidro Health Center, San Ysidro, USA

**Keywords:** Implementation, Integrated care, Pediatrics, Primary care, Mental health, Autism spectrum disorder

## Abstract

**Background:**

There is a critical need for effective implementation of integrated healthcare systems for children with autism spectrum disorder (ASD). Children with ASD have many service needs, including the need to access effective mental healthcare, given high rates of co-occurring psychiatric conditions. Pediatric primary care is an ongoing point of healthcare that is well positioned to identify mental health concerns and facilitate linkage to mental health services for children with ASD. However, identifying mental health problems in children with ASD by primary care providers is complex, subject to being overlooked and may significantly vary based on primary care organizational characteristics. Efforts targeting integrated primary-mental healthcare implementation require a tailored approach for children with ASD.

**Methods:**

This mixed methods, community-partnered study will apply the Exploration, Preparation, Implementation, Sustainment (EPIS) framework (Aarons et al., 2011; Moullin et al., in press) to adapt and implement an integrated care model, “Access to Tailored Autism INtegrated Care” (ATTAIN), in pediatric practices within three diverse healthcare settings for children ages 4–18 years. Key inner context factors from the Exploration, Preparation, and Implementation phases of the EPIS framework will guide three objectives of this study: (1) to identify targets to improve mental health screening and linkage to mental health services in primary care for children with ASD, (2) to adapt integrated care procedures to facilitate identification of mental health problems and linkage to evidence-based care for children with ASD, and (3) to examine feasibility, acceptability, and uptake of the adapted integrated mental healthcare model through a pilot study in pediatric primary care.

**Discussion:**

Improving integrated mental healthcare for children with ASD could have a significant public health impact on mental healthcare access, child clinical outcomes, and reduction in healthcare costs. Results from this mixed methods study will inform selection of implementation strategies to conduct larger-scale implementation of tailored integrated mental healthcare for children with ASD that will ultimately help to address the high unmet mental health needs for these children.

## Background

There is a critical need for effective implementation of integrated healthcare services for children and adolescents with autism spectrum disorder (ASD) whose numbers are rapidly growing [1]. Children with ASD often have complex physical and mental healthcare needs that necessitate both pediatric primary care and specialty care services [2, 3]. The prevalence of ASD and co-occurring psychiatric conditions (e.g., disruptive behaviors, attention and hyperactivity problems, anxiety) is estimated at greater than 70% [4, 5]. However, studies have shown that 41% of these children with ASD and co-occurring mental health conditions have substantial unmet mental healthcare needs [6]. Timely identification of co-occurring psychiatric conditions and linkage to mental health services for children with ASD is imperative to facilitate appropriate treatment and behavioral improvements [7].

Pediatric primary care is a principal point of care for children with ASD, so it is an ideal setting for early identification and ongoing monitoring of mental health needs and linkage to needed mental healthcare [8, 9]. Implementation of integrated mental healthcare is a potential solution to targeting timelier mental health screening and service linkage for children with a known diagnosis of ASD who also have co-occurring mental health conditions requiring intervention. Integrated care is an umbrella term that can encompass many different care arrangements. For this study, we define integrated care as a team of primary care providers and mental health specialists collaborating with the patient-family unit to coordinate care [10]. Although integrated care is not standard in pediatric primary care, emerging support exists for integrated healthcare approaches for children with ASD to facilitate addressing unmet specialty healthcare needs, including those for mental health [6, 11]. Further, when caregivers perceive themselves as shared decision-makers—an important component of integrated care—they report greater satisfaction with their child’s care quality [12]. Overall, integrated healthcare approaches show promise in reducing unmet mental health needs for children with ASD.

There are many patient, provider, organization, and system-level challenges to implementing integrated mental healthcare for ASD. One major challenge is diagnostic overshadowing. This is the process by which a diagnosis such as ASD obscures or prevents the diagnosis and treatment of other comorbid physical and/or mental health problems [13–18]. Another major challenge is the way pediatric primary care itself is structured, with well-child appointments typically lasting 11–20 min or less. Many topics need to be covered during the visit [19]. This places a significant burden on the pediatric primary care provider and caregiver to efficiently and effectively identify mental health concerns. In addition to limited time in pediatric primary care appointments, caregivers report many barriers to accessing pediatric specialty mental health services such as few qualified providers/services to treat children with ASD who have other psychiatric problems, inadequate care coordination and communication between pediatricians and other specialty providers, and difficulty obtaining information about specialized services [6, 20–22]. These implementation challenges suggest the potential need for increased ASD-specific mental health training of primary care providers to promote successful identification of co-occurring psychiatric conditions and initiate the integrated care process for children with ASD. Integrated care models are ideally suited to the way to address these barriers to mental healthcare for families of children with ASD who require additional assistance in accessing mental health services [23, 24].

To date, there have been no studies that have tested the use of integrated mental healthcare models for children with ASD within real-world pediatric primary care settings. This may be in part because many adaptations would need to be made for children with ASD. For example, screening instruments may need to be adapted or carefully selected to accurately identify mental health problems in children with ASD that may require targeted mental health treatment. This may be important because (1) core symptoms of ASD may overlap with other non-ASD psychiatric disorders (e.g., anxiety) and (2) non-ASD psychiatric disorders may manifest differently in children with ASD (e.g., for a child with ASD and anxiety, this may manifest as increases in repetitive or sensory behaviors versus rumination) [25]. Another key component of integrated care for children with ASD would be an integrated electronic health record to facilitate the coordination of the many services and healthcare providers necessary for effective treatment. This includes improved interoperability of the electronic health record to competently facilitate information sharing between providers [26]. Finally, there would need to be additional service navigation support, facilitated by the electronic health record and/or dedicated staff, for mental health screening and linkage for children with ASD, which has been strongly recommended to help families of children with ASD access appropriate treatment services [27].

The Access to Tailored Autism INtegrated Care (ATTAIN) study is designed to address the need for systematically adapting and testing an integrated care model for ASD. The ATTAIN study is a mixed methods implementation study that uses screening and linkage practices from the broader pediatric literature as well as the literature on adult integrated care to adapt the process of integrated care for children with ASD and co-occurring mental health conditions.

## Implementation science conceptual framework and approach

**Fig. 1:**
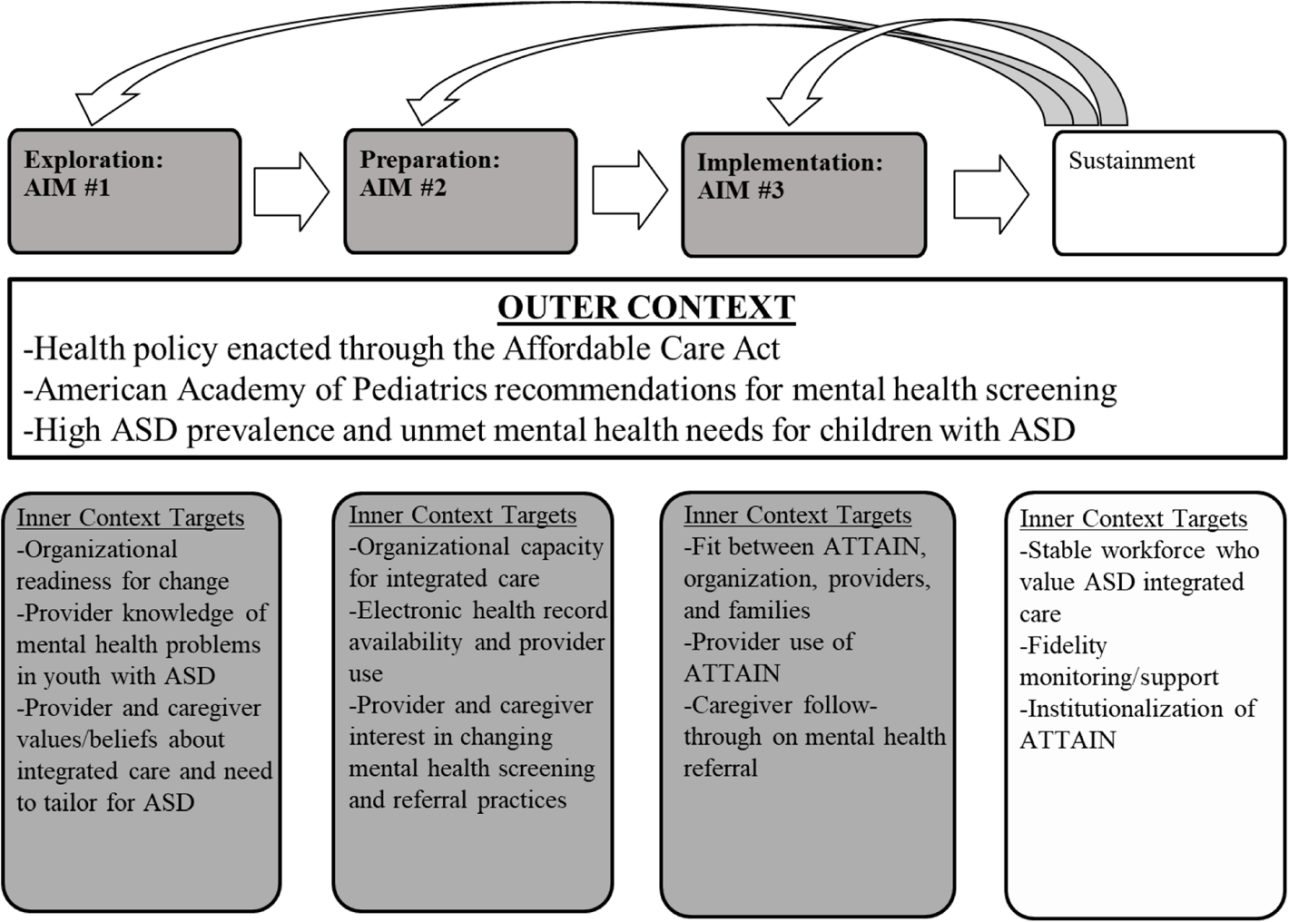
Application of the EPIS framework (adapted from [28]). Shaded components are the focus in this study

This study applies the Exploration, Preparation, Implementation, and Sustainment (EPIS) framework [28, 29] to identify specific contextual factors to target when implementing ATTAIN in primary care settings*.* The EPIS framework was selected because it is a four-phase, prospective implementation framework that defines outer (i.e., system-level) and inner (i.e., organizational, provider, patient) context factors that may influence implementation and sustainment of a new practice in a clinical setting. Figure 1 details the targeted outer and inner context factors across each EPIS phase that are relevant to implementation of ATTAIN in this study and broader pediatric integrated care implementation efforts. The outer context is conceptualized as stable across phases of ATTAIN implementation while the inner context evolves over the phases and represent the specific targets of the study. The first three phases (shaded in Fig. 1) are the focus of this study.

A key component within the EPIS framework and that is an essential implementation strategy within this study involves the inter-organizational relationships among stakeholders and entities. In this study, this is represented through community-academic partnership [30]. Specifically, community partnerships have been established with three primary care healthcare organizations in Southern California: (1) a linked health system with the largest pediatric primary care group in two Southern California counties, serving families with private insurance and Medicaid, (2) a private, for-profit integrated healthcare system that has a variety of payment systems including self-pay, employer-based private insurance, and subsidized programs, and (3) a federally qualified health center that serves a racially/ethnically/linguistically diverse and lower-income patient population along the US/Mexico border.

The “ATTAIN Advisory Group” will be developed to maximize the “fit” between ATTAIN and a range of pediatric primary care contexts. The ATTAIN Advisory Group includes stakeholders from the three healthcare organizations (described above) that serve children with ASD. The ATTAIN Advisory Group will be established at the outset of the study and meet regularly to guide study design choices, interpretation of results, implementation of findings in primary care settings, and evaluation of the collaborative process. The ATTAIN Advisory Group will include the principal investigator and approximately eight community stakeholders who represent pediatric and mental health providers, researchers, leaders, and caregivers of children with ASD.

Using key inner context factors from the EPIS framework, three objectives of this study will be pursued: (1) to identify targets to improve mental health screening and linkage to mental health services in primary care for children with ASD, (2) to adapt integrated care procedures to facilitate identification of mental health problems and linkage to evidence-based care for children with ASD, and (3) to examine feasibility, acceptability, and uptake of the adapted integrated mental healthcare model through a pilot study in pediatric primary care.

## Methods

The study design and methods are presented in the following sections by study aim.

### Aim 1

Aim 1 is to use a quantitative concurrent with qualitative (QUAN+QUAL) mixed methods design [31, 32] to identify targets to improve mental health screening and linkage to mental health services in primary care for children with ASD. Perspectives from organizational leaders, pediatric providers, and caregivers of children with ASD will be gathered to inform adaptations needed to tailor such practices for children with ASD.

#### Participants

In this study, the term “provider” indicates a practitioner or clinician, rather than an organization. Purposeful sampling will be used to maximize variation in perspectives and depth of information about mental health screening and linkage practices in primary care settings [33]. Twenty participants will be recruited from each of the three organizations (total *n* = 60). These participants will be organizational leaders in primary care (e.g., executive director, director of pediatrics department, primary care clinic lead), primary care providers (e.g., pediatricians, developmental behavioral pediatricians, nurse practitioners, case managers), and caregivers of children with a known ASD diagnosis who receive primary care at one of the three healthcare organizations. This target sample size was selected based on recommendations from mixed methods implementation and qualitative method research [33, 34] to pursue the mixed methods functions of convergence, complementarity, and expansion and achieve *a priori* thematic saturation (based on the EPIS framework).

#### Procedure

Recruitment will occur through several methods including in-person appearances at staff meetings and flyers posted in patient areas. Those who are interested in study participation will be asked to complete a study contact form that includes their preferred contact details to receive more information about the study activities (focus group and survey). Focus groups will occur on-site at each organization or at a convenient location. At least three focus groups per organization will be conducted based on participant type: (1) organizational/clinic/program leadership, (2) pediatric providers, (3) caregivers of children with ASD. Focus groups will take approximately one hour and participants will receive a $40 gift card for their participation. Surveys will be distributed using web-based software. The survey will take approximately 20 min to complete, and participants will receive a $20 gift card for their time.

#### Instruments

Data will be collected using a web-based survey (QUAN) and focus groups (QUAL). The content of the survey and focus groups will be designed to elicit data gathering about key inner targets from the Exploration and Preparation phases of the EPIS framework [28] shown in Fig. 1. There will be two versions of the web-based survey: primary care provider and caregiver. The provider/staff version will include items from the Geisinger Health System’s Primary Care Physician needs assessment survey [35] regarding current use of mental health screening and comfort identifying mental health problems in children with ASD. The caregiver version will include items from the National Survey of Children with Special Health Care Needs, Access to Care Questionnaire [36] related to experiences in coordinating and accessing mental health services. These surveys are available upon request.

There will be two versions of the focus group guide: primary care provider and primary care leader. The focus group guides will be designed to support a semi-structured approach to include pre-selected interview questions to promote active participation of participants who have experience with or directed perspectives on mental health screening practices in their pediatric primary care setting for children with ASD [37, 38]. Probes will be used as needed to explore issues in more depth. Focus group content was finalized with consultation from the ATTAIN Advisory Group and includes the following constructs: the mental health needs of children with ASD served in primary care, comfort discussing mental health in primary care appointments, current mental health screening and linkage to evidence-based mental health procedures, and needed adaptations to current procedures for children with ASD. These focus group guides are available upon request.

#### Analysis

Mixed-methods analytic approaches will be used to integrate the data and results across methods and used to examine convergence (i.e., do the two methods confirm or find similar results?), complementarity (i.e., do the two methods provide more depth of understanding of research questions?), and expansion (i.e., do the two methods provide insights beyond either method alone?) [31]. Both data methods will be triangulated to analyze convergence, to understand whether qualitative and quantitative data yield similar responses to specific questions. Finally, as recommended by Aarons and colleagues [31], qualitative and quantitative analyses will be integrated to examine whether focus group data contextualize targets assessed in survey data for the purpose of expansion [31, 32].

### Aim 2

Aim 2 is to adapt integrated care procedures to facilitate identification of mental health problems and linkage to evidence-based care for youth with ASD. The design of this aim is to capitalize on the collaborative process of the ATTAIN Advisory Group to use the findings from the first aim to refine the components of ATTAIN and prepare for the pilot trial in the subsequent and final aim of the study.

#### Participants

The ATTAIN Advisory Group will participate and aid in finalizing components of ATTAIN. The members of the ATTAIN Advisory Group include the principal investigator and approximately eight stakeholders who are pediatric and mental health providers, researchers, or leaders and caregivers of children with ASD.

#### Procedure

Results from the mixed methods needs assessment from the first aim will be discussed during a series of meetings of the ATTAIN Advisory Group to guide identification of adaptations and co-develop refinements needed for the ATTAIN model. The possible adaptations to the standard integrated health approaches might include (1) administration of a pre-appointment, mental health screening instrument such as the Pediatric Symptom Checklist-17 [39–42] that may need adaptation to effectively identify manifestation of mental health symptoms within the context of an ASD diagnosis, (2) pediatric provider training to interpret and briefly discuss the screening results and rationale for a mental health referral over and above existing treatment services that the family may be receiving, (3) use of the electronic health record to facilitate a mental health referral for children with ASD who present with clinically elevated scores on the mental health screener, (4) tailored service navigation assistance to link families of children with ASD to mental healthcare, (5) access to evidence-based care (internal or external to the referring healthcare setting) that targets co-occurring mental health problems within the context of an ASD diagnosis, (6) electronic health record tracking of mental health appointment completion, and (7) collaboration between primary care and mental health providers for ongoing care. Meetings of the ATTAIN Advisory Group will occur on an approximately monthly basis, each lasting 1–2 h over 6 months. During each meeting, the ATTAIN Advisory Group will engage in a group discussion to review and refine drafted materials related to the ATTAIN model and implementation supports needed for the subsequent pilot study (Aim 3) in partner organizations. In these meetings, there will also be guided discussion about areas of the ATTAIN model and implementation supports that may require tailoring for primary care or mental healthcare settings (e.g., identifying alternative mental health referral mechanisms if an organization’s electronic health record limits changes to workflow structures)**.**

#### Measures

The collaborative process will be assessed by the ATTAIN Advisory Group Collaborative Process Survey (AIM Study; PI: Brookman-Frazee; [43]) that will be administered at the conclusion of each group meeting.

### Aim 3

Aim 3 is to conduct an open trial feasibility pilot test of ATTAIN (directly informed by adaptations identified in Aim 2) in pediatric primary care offices. The primary implementation outcomes that will be measured are ATTAIN feasibility, acceptability, uptake, and speed of implementation. In addition, two primary service outcomes will be measured: mental health services access and communication between primary care and mental health provider.

#### Participants

A total of 60 primary care providers will be recruited from primary care practices in the region. It is expected that 45 providers will agree to participate. This targeted sample size was chosen based on sample sizes used within completed pilot studies focused on service interventions in pediatric primary care [42]. Each provider will be asked to use ATTAIN with their pediatric patients who have a documented ASD diagnosis in the electronic health record over four consecutive months.

#### Procedure

Leadership at each primary care organization will be approached about their primary care practices participating in the pilot study. Primary care practices within each organization will be purposively selected for targeted recruitment of providers for ATTAIN implementation based on patient volume. Training in ATTAIN delivery will take place in advance of active implementation of ATTAIN. Subsequently, trained staff and providers will be asked to use ATTAIN with patients who have a documented ASD diagnosis during any outpatient appointment (e.g., annual check-ups, drop-in appointments) to maximize early detection of mental health problems and opportunity to link to mental health services. Quantitative data of ATTAIN use will be extracted from patient charts. After 4 months of ATTAIN delivery, participating providers and caregivers of youth with ASD with whom providers used ATTAIN will be asked to complete a brief online survey regarding their experiences using ATTAIN. Providers and staff will receive a $100 honorarium and caregivers will receive $20 for survey completion.

#### Measures

##### Feasibility and acceptability of ATTAIN

The Perceived Characteristics of Intervention Scale (PCIS) [44] will be used to examine providers perspectives regarding feasibility and acceptability of ATTAIN. The PCIS is a 20-item scale that assesses attitudes towards a specific intervention including relative advantage, compatibility, and complexity. Participants are asked to rate the extent to which they agree with each item on a 5-point Likert scale. Example items include, “The ATTAIN model is clear and understandable” and “Using the ATTAIN model fits well with the way I like to work.” Caregiver perspectives of feasibility and acceptability of ATTAIN will be measured using modified items from the caregiver web-based survey administered in Aim 1 and drawn from the National Survey of Children with Special Health Care Needs, Access to Care Questionnaire [36] .

##### Uptake

ATTAIN uptake will be measured using extracted data from the electronic health record system. Specifically, uptake will be defined as the proportion of ATTAIN-eligible patients (i.e., children with a documented ASD diagnosis) to the number children with whom providers used ATTAIN. Providers will be asked to self-report the patients with whom they used ATTAIN.

##### Speed of implementation

The Stages of Implementation Completion [45] will be adapted to assess implementation progress of ATTAIN. This assessment tool includes eight stages that occur within three phases of implementation (pre-implementation, implementation, and sustainability). Scores are calculated for the speed of implementation as indicated by the amount of time spent in each stage, and the proportion of implementation activities completed.

##### Service outcomes

Mental health services access will be measured by documentation of successful completion of the first mental healthcare appointment and the proposed evidence-based practice that the mental healthcare provider will use. Communication between the referred-to mental health provider and referring primary care provider will be measured by documentation of a consultative interaction regarding the specified patient. These data will be extracted from the electronic health records of the children with whom providers reported using ATTAIN.

##### Covariates

Provider and caregiver sociodemographic variable will be available from the post-ATTAIN use online surveys. For child covariates, the following will be extracted from the electronic health records of children with whom providers used ATTAIN: sociodemographic characteristics, funding source for primary and mental healthcare, and documented mental health diagnoses, if any.

#### Analysis

Descriptive statistics will first be conducted to characterize patterns of feasibility, acceptability, and uptake of ATTAIN over 4 months across and between the organizations that participated. Analyses of variance (ANOVA) will subsequently be performed to identify significant group differences in outcome measures between participating organizations. Additionally, the distribution of and correlations among provider, caregiver, and child covariates and outcome measures will be examined. Based on significant bivariate associations, subsequent linear regression models will be performed to identify predictors of the primary implementation outcomes. Given the pilot study design with explicit focus on feasibility, effect size estimation based on commonly used guidelines [46] will be emphasized.

## Discussion

### Innovation

This mixed methods study aims uses the EPIS model [28] and a community-partnered approach to adapt and implement an integrated mental healthcare model for children with ASD and other mental health needs, ATTAIN, in pediatric primary care. This study offers several important innovations that contribute to the field of implementation science. First, it capitalizes on existing knowledge about integrated care and available electronic health record capabilities in healthcare systems to *adapt* components of mental health screening and linkage specifically for children with ASD for implementation in primary care. By adapting (versus developing new), the ATTAIN model offers the potential to accelerate implementation efforts and mental health service access for children with ASD who are a unique, high need group. Another innovative component is the clearly specified application of the EPIS implementation framework to guide identification of specific targets (e.g., provider knowledge of identifying mental health problems in children with ASD, organizational support to adapt screening practices) of ATTAIN implementation that are hypothesized to shape earlier identification of mental health problems and linkage to necessary mental healthcare (outcomes). Second, this study’s approach and implementation in pediatric primary care is guided by a community-academic partnership that includes stakeholder representatives from three diverse organizations that provide primary care to children with ASD. Employing a community-academic partnership that includes collaboration with pediatric primary care and mental health leaders, providers, and consumers (i.e., caregivers of children with ASD) from multiple healthcare organizations that differ in organizational (inner context) characteristics may optimize the reach and sustainment of ATTAIN.

### Limitations

Some methodological limitations warrant note. This study intentionally focuses on children with co-occurring ASD and non-ASD mental health conditions. It also comprises a developmental pilot study. The purpose of this design and approach is to systematically identify adaptations for implementation that can be tested in larger-scale trials and inform clinical implementation expansions to broader pediatric groups. As a result, this study is not statistically powered for traditional significance testing. However, effect size estimation [46] will be emphasized when interpreting quantitative study findings and is a more appropriate approach for pilot studies of feasibility.

Another limitation is the quasi-experimental study design. While this design is appropriate given the primary purpose of assessing the feasibility of ATTAIN implementation, the lack of randomization limits the extent of interpretation. One methodological decision to address this is capitalizing on variance vis-à-vis inclusion of three primary care organizations to assess implementation feasibility across diverse healthcare settings. Additionally, the inclusion of a community-academic partnership is a methodological advantage because community-academic partnership members can contextualize findings through their specialized organizational knowledge, which may bolster the clinical and implementation significance of results. Despite these limitations, this study has potential to further the field of integrated healthcare for children with ASD by accelerating identification of mental health problems in primary care and access to mental healthcare that may ultimately optimize child functioning and reduce expenditures.

### Impact

The healthcare landscape for children with ASD is fragmented, challenging to navigate, and contributes to these children’s unmet mental health needs. Improving mental health screening and linkage to care for this population could have a significant public health impact on mental healthcare access, child clinical outcomes, and reduced healthcare costs. Identifying adaptations required to facilitate successful efforts in integrating primary and mental healthcare for children with ASD and implementing these adaptations is critical to mitigate the unmet mental health needs for this group. The findings yielded from this study will inform a large-scale hybrid implementation effectiveness trial of ATTAIN. Although the current study is focused on implementation of the ATTAIN model, an adapted integrated care model for children with ASD, ATTAIN is an entry point for improving care that may be generalizable to other clinical populations who experience under-identification of mental health problems (e.g., children with chronic healthcare conditions).

### Trial status

The University of California, San Diego, as well as each healthcare organization’s Institutional Review Board or equivalent has approved the study procedures. At the time of the original submission of this manuscript (May 2018), we have started enrollment of caregivers, providers, and organizational leaders for Aim 1 data collection.

## Abbreviations

ASD: Autism spectrum disorder
ATTAIN: Access To Tailored Autism INtegrated Care
EPIS: Exploration, Preparation, Implementation, Sustainment
